# Uterine dynamics, blood profiles, and electronic fetal monitoring of primiparous and multiparous bitches classified according to their weight

**DOI:** 10.3389/fvets.2023.1282389

**Published:** 2023-11-16

**Authors:** Karina Lezama-García, Julio Martínez-Burnes, Uri Baqueiro-Espinosa, Dina Villanueva-García, Adriana Olmos-Hernández, Ismael Hernández-Ávalos, Patricia Mora-Medina, Adriana Domínguez-Oliva, Daniel Mota-Rojas

**Affiliations:** ^1^PhD Program in Biological and Health Sciences, Doctorado en Ciencias Biológicas y de la Salud, Universidad Autónoma Metropolitana, Mexico City, Mexico; ^2^Facultad de Medicina Veterinaria y Zootecnia, Universidad Autónoma de Tamaulipas, Ciudad Victoria, Tamaulipas, Mexico; ^3^School of Biological Sciences, Queen’s University Belfast, Belfast, United Kingdom; ^4^Division of Neonatology, Hospital Infantil de México Federico Gómez, Mexico City, Mexico; ^5^Division of Biotechnology-Bioterio and Experimental Surgery, Instituto Nacional de Rehabilitación Luis Guillermo Ibarra Ibarra, Mexico City, Mexico; ^6^Facultad de Estudios Superiores Cuautitlán, Universidad Nacional Autónoma de México, Cuautitlán Izcalli, Mexico; ^7^Neurophysiology, Behavior and Animal Welfare Assesment, DPAA, Universidad Autónoma Metropolitana, Mexico City, Mexico

**Keywords:** uterine contractions, bitch parturition, fetal monitoring, hypoxia, blood profile

## Abstract

Perinatal mortality occurs in all species. In dogs, mortality rates have been reported to range from 5 to 35%. Electronic fetal and uterine monitoring has recently been used in domestic animals to monitor the mother and newborn before and during parturition. In this way, the fetal heart rate and uterine dynamics can be monitored. This study evaluated the uterine dynamics of bitches with different weights and parity. Ninety-six bitches and their 476 puppies were divided into four experimental groups containing 24 individuals each (12 primiparous bitches and 12 multiparous bitches), according to body weight: G_1_ (4–8 kg), G_2_ (8.1–16 kg), G_3_ (16.1 to 32 kg), and G_4_ (32.1 to 39.6 kg). The fetal heart rate decelerations (dip 2 patterns), uterine dynamics, and bitches’ blood profiles were evaluated, including levels of glucose, lactate, pCO_2_, pO_2_, pH, HCO_3_^−^, and Ca^++^. The dam weight can affect the vitality of newborns and the uterine dynamics, with differences in the frequency, intensity, and duration of myometrial contractions. The expulsion interval between puppies was longest in primiparous bitches with low weight and shortest in multiparous bitches with high weight. The expulsion interval and the number of stillborn females were higher in primiparous bitches with high weight. Newborn male puppies were significantly heavier than newborn females.

## Introduction

Birth is a physiological process in which the fetus is expelled from the uterus through uterine contractions and cervical dilation (1). These contractions are caused by the release of various hormones, including oxytocin (1), and by changes that cause depolarization and repolarization of the myometrial cells (2).

In veterinary perinatology, high prenatal, intrapartum, and perinatal mortality is observed in some domestic species. Therefore, to reduce these high rates, it is essential to monitor the development of the fetus throughout pregnancy and parturition, which can improve perinatal care and reduce newborn mortality (3, 4). For example, in dogs (*Canis lupus familiaris*), mortality rates of 17–30% have been reported (5), although, according to Veronesi et al. (6), these rates may range from 5 to 35%.

Pregnancy in dams usually lasts 63 days ±1 day (7). Parturition consists of 3 phases: Stage 1 is characterized by intermittent uterine contractions associated with cervical dilation and behavioral changes (1); Stage 2 includes intensified uterine contractions accompanied by abdominal efforts and the Ferguson reflex, which produces fetal expulsion; and finally, Stage 3 is characterized by placental expulsion (8).

Electronic fetal and uterine monitoring is one of the main methods used to clinically assess and determine the vitality and welfare of fetuses and the uterine dynamics of the mother before and during parturition (9–12). In this sense, fetal and uterine electronic monitoring records fetal movements, the fetal heart rate (in bpm, bpm), and uterine contractions (in mmHg) (13). The second component of a fetal and uterine monitor is the tocodynamometer. This device measures uterine contraction intensity, frequency, and duration (14). It is a practical, noninvasive commercial alternative to traditional Doppler techniques (3) that is safe for the fetus and the mother (15, 16). Davidson (8), Groppetti et al. (17), Ayres-De-Campos and Nogueira-Reis (11), and Lezama-García et al. (4) have recently implemented this monitoring technique in bitches because in this species, it helps prevent and reduce mortality before, during and after parturition. In bitches (*Canis lupus familiaris*), this technique can detect dystocia, facilitating the prediction of whether a birth will end in a cesarean section (18), thereby allowing the timely detection of problems that cause fetal stress and pathological conditions, such as hypoxia and metabolic acidosis (11). In addition, it is a tool that can be used at home (e.g., by a breeder) with previous training. Dogs tolerate it well since it is not invasive, and the use of this tool can considerably decrease anxiety around the birth for the person responsible for the animal (8).

Electronic fetal and uterine monitoring parameters, such as heart rate, waveform, and dynamics of fetal behavior, are essential in determining fetal life, development, and maturity and detecting fetal stress or congenital heart disease (10, 19). The recording is carried out on the dam’s abdominal skin with an ultrasound transducer, which detects the fetal heart rate, and a pressure transducer, which evaluates the activity of the uterus; both devices are connected to a screen where the data can be observed. The results are printed on millimetric paper (12).

The fetal heart rate (FHR) is one of the most critical parameters used to determine the health and welfare of a fetus. By monitoring this variable, we can detect oxygenation failures in a timely manner (19), thus avoiding fetal hypoxia (20, 21) and possible secondary neurological damage or even death during birth (10). The FHR is influenced by the autonomic nervous system, and the level of these responses depends, in turn, on the amount of oxygen the fetus has access to (11). Therefore, when oxygen levels of the fetus drop sharply, an immediate FHR fall occurs (22). A sustained deceleration in the FHR reflects distress in the fetus. Therefore, it is essential to know the normal parameters of canine and feline FHR at the end of pregnancy, which are 170–230 beats/min or at least four times the maternal heart rate (20, 23).

The present study aimed to evaluate the uterine dynamics of bitches with different weights and parity. Our research questions were as follows: What is the effect of the weight of the bitch at parturition on the intensity, frequency, and duration of contractions at the expulsion phase of parturition? Are there differences in the intensity, duration, and frequency of uterine contractions between primiparous and multiparous bitches? Are the birth weight and expulsion interval of pups essential factors for predicting their survival? Finally, does the sex of the newborn influence its survival?

## Materials and methods

### Facilities

The present study was carried out in six veterinary clinics and hospitals in the City of Campeche, Campeche, located in southeastern Mexico, within the Yucatan peninsula. Authorization was requested from the owners to allow the veterinary clinics to care for their bitches throughout gestation, from 28 days after mating until 48 h postpartum.

### Study population

One hundred thirteen pregnant bitches between 2 and 6 years of age, primiparous or multiparous (1–4 previous litters), were recruited. However, since 17 of these bitches (five with primary uterine inertia and 12 with secondary uterine inertia) had dystocic parturition that ended in cesarean section or required the supply of oxytocin and calcium, they were excluded from the study. Therefore, a total of 96 bitches were finally included, with 476 puppies. The 96 dams were divided into four experimental groups containing 24 individuals each (12 primiparous and 12 multiparous), according to their body weight: G_1_ (4–8 kg), G_2_ (8.1–16 kg), G_3_ (16.1 to 32 kg), and G_4_ (32.1 to 39.6 kg). Body weight was obtained at the onset of contraction, when the first whelping stage started, through a digital scale (Avery Weigh-Tronix 7,820–100 West Bromwich, UK). The breeds included in this study were as follows: Chihuahua, Yorkshire Terrier, Cocker Spaniel, Standard Schnauzer, Scottish Terrier, Miniature Poodle, German Shepherd, Labrador, Golden Retriever, Great Dane, and Belgian Shepherd. The inclusion criteria were as follows: (a) clinically healthy bitches receiving preventive medicine (e.g., vaccination/deworming protocols), (b) no clinical records of reproductive problems, and (c) bitches that had undergone ultrasonographic and radiographic studies to confirm natural whelping. Animals with the following characteristics were excluded from this study: (a) records of dystocia or pyometra, (b) malformed fetuses, (c) requiring the administration of birth inducers or accelerators, (d) aggressive individuals, (e) a body condition over 8 (obese: ribs not palpable under hefty fat cover, or palpable only with significant pressure; heavy fat deposits over lumbar area and base of tail, waist absent, no abdominal tuck, apparent abdominal distention) as per the WSAVA scale (24), (f) brachycephalic breeds known to have a high incidence of dystocia, or (g) receiving an emergency C-section (25). The body weight ranges were based on those of the Federation Cynologique Internationale (FCI): small (dogs up to 30 cm in height and 15 kg in weight), medium (dogs between 30 and 40 cm in height and between 15 and 25 kg in weight), and large (dogs between 40 and 60 cm in height and between 25 and 45 kg in weight) (26). Type I stillbirths (SBs) were excluded from the study (a total of five type I stillbirths were observed), and only type II stillbirths were included, classified by necropsy. According to Mota-Rojas et al. (27, 28), SBs can be classified as type I (prepartum or antepartum deaths), which are deaths before the end of gestation due to infectious causes; fetuses appear haemorrhagic, oedematous, and have grayish-brown discolouration. Type II SBs (intrapartum deaths) refer to deaths during whelping due to intrauterine asphyxia and are rarely caused by infectious diseases; puppies maintain a normal appearance similar to their littermates but lack respiration.

### Clinical history

In the clinics and hospitals where the study was carried out, standardized veterinary software (SmartZooft® LAN version 14 K, developed by SQUENDA®, Mexico City, Mexico) was used, and the clinical history was recorded. Such data included age, breed, type of diet, parity, body weight, preventive medicine history, and address, as well as the general data of the owner.

### Prenatal procedures

All females included in the study became pregnant through direct mating. At 28 to 30 days after mating, pregnancy was confirmed with Mindray® model DP-30VetPower ultrasonography equipment (Shenzhen, China) with Doppler and pulsed Doppler (PW) utilizing a 3.5 MHz convex transducer. Gestation was confirmed by visualization of the gestational sacs and embryo heartbeat. Another ultrasonographic assessment was performed between 40 and 43 gestational days to verify the health, growth, and vitality of fetuses. Since mating or insemination cannot be used as indications of pregnancy (29–31), gestational age was confirmed through ultrasonography (7). Gestational age was determined by applying the following formulas: GA = DGS × 6 + 20 ± 3 days for gestations shorter than 40 days or GA = BPD × 15 + 20 ± 3 days for gestations longer than 40 days, where BPD is the biparietal diameter and DGS is the diameter of the gestational sac (32).

On days 48–50 after mating, X-rays were performed to identify and exclude bitches with possible dystocia (due to cephalopelvic disproportion) (33), determine the number of fetuses, and evaluate the size of their heads.

**Figure 1:**
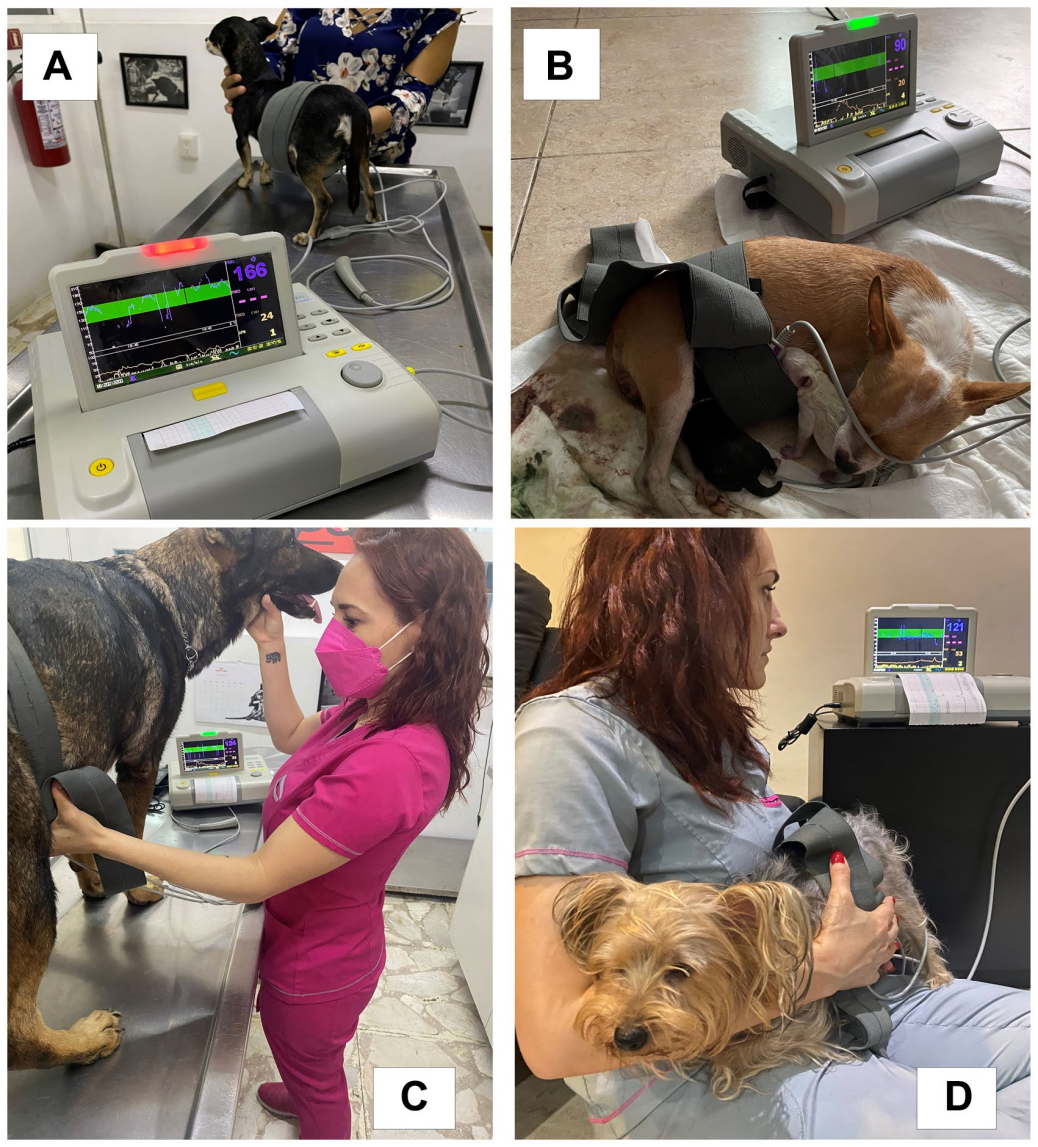
Electronic uterine and fetal monitoring. **(A)** Electronic uterine and fetal monitoring during parturition in a small multiparous bitch. **(B)** Monitoring of a Chihuahua dam during parturition. **(C)** Monitoring of a Belgian Shepherd dam. **(D)** Monitoring of a Yorkshire Terrier dam at the beginning of parturition. As shown in these images, monitoring was not invasive or painful, and the bitches allowed the veterinarian to place the adjustable bands that hold the tocodynamometer and the transducer on the skin of the abdominal zone.

On day 60 after mating, the fetuses and bitches were monitored using a Sonolife® (Chihuahua, Mexico) brand antepartum monitor, Smart Monitor Color model, with a multicrystal pulsed Doppler transducer. The monitor assessed the health status of both the bitch and the fetuses, the fetal heart rate, and uterine activity, including the number, duration, interval, and frequency of the contractions, following a methodology previously reported for use in piglets by other authors (34) (Figures 1A–D). It is worth mentioning that the assessment of uterine dynamics was carried out from the moment that the chorioallantois membranes could be observed in the vulva; this time point was considered the beginning of the expulsion phase of whelping, which was monitored for the first 60 min. The expulsion phase duration was defined as the period from when the chorioallantois membranes were observed in the vulva until the puppy was entirely expelled.

Decelerations of FHR, specifically dip 2 patterns (a drop in the FHR beginning after the onset of a uterine contraction and returning to baseline after the uterine contraction has ended, caused by uteroplacental insufficiency) were also identified to determine the degree of fetal asphyxia *in utero*. These changes (dip 2) are attributed to the transitory occlusion of umbilical vessels due to the contracted uterus. According to Vispo et al. (35), fetal hypoxia develops when the occlusion is short and lasts less than 40 s. Following Mota-Rojas et al. (36), unfavorable dip 2 patterns were considered if they lasted more than 60 s and when the FHR was less than 70 bpm. To clinically detect these unfavorable dip 2 patterns, the FHR was evaluated before, during, and immediately after the myometrial contraction, and observations were carefully recorded when this coincided with the contraction’s peak. When dip 2 patterns arose, an emergency cesarean section was performed, and these bitches were excluded from the analysis, which is why none of the puppies included in this study presented meconium-stained amniotic fluid.

The monitoring of the vital signs of the dams was carried out using a veterinary monitor DESEGO® (Mexico City, Mexico) Model M8i SVGA to evaluate the electrocardiographic tracings, respiratory rate, oxygen saturation, temperature, and blood pressure from the probable date of whelping. Fetal heart rate was monitored before and during labor using the uterine and fetal electronic monitor described above.

### Neonatal procedures

The newborn’s heart rate was evaluated with a 3M™ Littmann classic pediatric III-5620 stethoscope (Canada). Neonatal heart rates below 100 bpm were considered to indicate bradycardia. Puppies were weighed with a digital scale (Salter Weight Tronix Ltd., West 148 Bromwich, UK). Rectal temperature was measured with a Hergom-Medical™ digital veterinary thermometer (Monterrey, Mexico). Puppies with rectal temperatures below 36°C were considered hypothermic.

### Blood sampling and blood profile analysis

Blood samples were collected from bitches via a puncture in the plantar pad, using microcapillary tubes impregnated with lithium, at the end of the 60 min of electronic monitoring. This time point was selected because it was easier to obtain the sample after dams had given birth and minimized disturbance. Samples were analyzed with GEM Premier™ 3,000 (Instrumentation Laboratory Diagnostics, Lexington, KY, USA/Milano, Italy) to obtain glucose (mg/dL), lactate (mg/dL), pCO_2_ (mmHg), pO_2_ (mmHg), pH, HCO_3_^−^ (mmol/L), and Ca^++^ (mmol/L) values.

### Statistical analysis

Analyses were performed in R version 4.2.2 (R Core Team, Vienna, Austria) using the packages “moments,” “ggpubr,” “stats,” “emmeans” and “multcompView.” The significance threshold was set at *p* < 0.05. ANOVA assumptions of normality and homoscedasticity were checked by visual inspection of model residuals using Q-Q plots and plots of residuals vs. predicted values. The results are presented as the mean ± SEM.

As shown in Table 1, two-way ANOVA was applied to assess the effect of parity and weight group on the expulsion phase duration, expulsion interval, and puppy birth weight. *Post hoc* pairwise comparisons were conducted with Tukey HSD tests. Differences in the proportion of stillborn puppies between primiparous and multiparous bitches classified according to their weight were detected with a chi-square test.

**Table 1:** Reproductive performance of primiparous and multiparous bitches at the expulsion phase of whelping classified according to their weight (mean ± SEM).

	G_1_		G_2_		G_3_		G_4_	
	*P*	*M*	*P*	*M*	*P*	*M*	*P*	*M*
Expulsion phase duration (min)	268.0 ± 31.3^b,c^	226.0 ± 18.3^c^	288.0 ± 10.2^b,c^	326 ± 20.1^a,b,c^	303.0 ± 32.0^b,c^	300.0 ± 17.8^b,c^	426 ± 36.0^a^	335.0 ± 18.9^a,b^
Expulsion interval between puppy	82.2 ± 4.8^a^	66.9 ± 3.2^b,c^	75.1 ± 3.01^a,b^	62.5 ± 2.5^b,c^	69.1 ± 3.9^a,b^	53.9 ± 2.4^c,d^	64.6 ± 2.8^b,c^	46.3 ± 1.3^d^
Stillborn puppies	7 (9.72%)	5 (6.94%)	9 (12.5%)	6 (8.33%)	10 (13.88%)	7 (9.72%)	17 (23.61%)	11 (15.27%)
Birth weight (g)	186.0 ± 4.8^d^	198.0 ± 2.8^d^	257.0 ± 6.6^c^	273.0 ± 2.9^c^	354.0 ± 3.4^b^	376.0 ± 6.8^a,b^	390.0 ± 10.6^a^	387.0 ± 4.5^a^

Two-way ANOVA/Chi-square test for the rate of stillbirths. Dams were divided into groups according to weight: G_1_, 4–8 kg; G_2_, 8.1–16 kg; G_3_, 16.1–32 kg; G_4_, 32.1–39.6 kg. P, primiparous; M, multiparous. ^a,b.c,d^Different superscripts across columns indicate statistically significant differences between groups (*p* < 0.05).

As shown in Table 2, differences in the intensity, duration, and number of contractions between primiparous and multiparous bitches classified according to their weight were detected with two-way ANOVAs. As the interval between myometrial contractions did not meet the normality or homoscedasticity assumptions, this variable was analyzed using a generalized linear model (GLM) with a “gamma” family distribution and “identity” link function. The predictors were weight group (4 levels), parity (2 levels), and their two-way interaction. *Post hoc* pairwise comparisons were conducted with Tukey HSD tests.

**Table 2:** Number, intensity, duration, and interval of myometrial contractions in primiparous and multiparous dams classified according to their weight (mean ± SEM).

	G_1_		G_2_		G_3_		G_4_	
	*P*	*M*	*P*	*M*	*P*	*M*	*P*	*M*
Intensity (mm/Hg)	35.1 ± 0.9^a^	30.1 ± 0.6^b^	30.2 ± 0.3^b^	24.8 ± 0.6^c,d^	28.6 ± 0.6^b^	23.8 ± 0.4^d^	27.7 ± 0.9^b,c^	23.2 ± 0.8^d^
Duration (sec)	175.0 ± 9.4^a^	122.0 ± 4.8^b^	165.0 ± 7.7^a^	120.0 ± 5.1^b^	171.0 ± 7.2^a^	116.0 ± 4.01^b^	172.0 ± 7.8^a^	118.0 ± 4.3^b^
Contractions number	12.1 ± 0.4^e^	9.0 ± 0.2^f^	14.8 ± 0.4^d^	11.2 ± 0.4^e^	18.7 ± 0.3^b^	14.9 ± 0.3^c,d^	20.7 ± 0.3^a^	16.7 ± 0.5^c^
Interval between contractions (min)	5.81 ± 0.1^b^	7.75 ± 0.2^a^	4.70 ± 0.1^c^	6.24 ± 0.1^b^	3.71 ± 0.1^d,e^	4.66 ± 0.1^c^	3.35 ± 0.1^d^	4.18 ± 0.1^c,e^

Two-way ANOVA/GLM with a gamma family distribution for the interval between contractions. Dams were divided into groups according to their weight: G_1_, 4–8 kg; G_2_, 8.1–16 kg; G_3_, 16.1–32 kg; G_4_, 32.1–39.6 kg; P: primiparous; M: multiparous. ^a,b.c,d,e^Different superscripts across columns indicate statistically significant differences between groups (*p* < 0.05).

As shown in Table 3, a GLM with a Poisson family distribution and “log” link function was used to investigate the effect of weight group and parity on the number of dip 2 patterns, number of puppies with after birth (AB) bradycardia, number of puppies with cyanotic oral mucosa, and number of hypothermic and adynamic pups per litter (dam). Weight group (4 levels), parity (2 levels), and their interaction were included as predictors. The number of puppies in each litter (litter size) was included as a covariate because larger litters tend to contain puppies with any of the following conditions: bradycardia (less than 100 bpm), cyanotic oral mucosa, hypothermia (less than 36°C) or adynamia.

**Table 3:** Fetal heart rate decelerations (dip 2) in fetuses and newborn puppies, number and percentage of bradycardic, cyanotic, hypothermic and adynamic puppies of primiparous and multiparous bitches classified according to their weight.

	G_1_		G_2_		G_3_		G_4_	
	P *n* = 39	M *n* = 41	P *n* = 47	M *n* = 63	P *n* = 53	M *n* = 67	P *n* = 79	M *n* = 87
DIP 2	11 (1.307 ± 0.3)	5 (0.565 ± 0.2)	8 (0.789 ± 2)	4 (0.294 ± 0.1)	11 (0.962 ± 0.2)	7 (0.484 ± 0.1)	14 (0.821 ± 0.2)	7 (0.373 ± 0.1)
Number of puppies with AB bradycardia	11 (28.2%) (1.307 ± 0.3)	7 (17%) (0.791 ± 0.2)	11 (23.4%) (1.084 ± 0.3)	6 (9.5%) (0.441 ± 0.1)	10 (18.8%) (0.874 ± 0.2)	6 (8.9%) (0.415 ± 0.1)	18 (22.7%) (1.056 ± 0.2)	10 (11.4%) (0.533 ± 0.1)
Number of puppies with cyanotic oral mucosa	12 (30.7%) (1.426 ± 0.4)	6 (14.6%) (0.678 ± 0.2)	13 (27.6%) (1.281 ± 0.3)	8 (12.6%) (0.588 ± 0.2)	13 (24.5%) (1.136 ± 0.3)	9 (13.4%) (0.622 ± 0.2)	24 (30.3%) (1.408 ± 0.2)	14 (16%) (0.746 ± 0.1)
Number of hypothermic and adynamic pups	14 (35.8%) (1.663 ± 0.4)	9 (21.9%) (1.017 ± 0.3)	14 (29.7%) (1.380 ± 0.3)	9 (14.2%) (0.662 ± 0.2)	18 (33.9%) (1.573 ± 0.3)	13 (19.4%) (0.899 ± 0.2)	23 (29.1%) (1.349 ± 0.2)	16 (18.3%) (0.852 ± 0.2)

The results are presented as the number (proportion ± SE). Dams were divided into groups according to their weight: G_1_, 4–8 kg; G_2_, 8.1–16 kg; G_3_, 16.1–32 kg; G_4_, 32.1–39.6 kg. P, primiparous; M, multiparous. n, number of puppies. AB, after birth.

As shown in Table 4, differences in the birth weight and expulsion interval between male and female puppies classified according to dam weight group were analyzed with separate two-way ANOVAs. The predictors were the weight group, sex of the puppy, and their two-way interaction. *Post hoc* pairwise comparisons were performed using Tukey’s HSD tests.

**Table 4:** Mean and standard error of birth weight and expulsion interval between females and males born from dams classified according to their weight.

	G_1_		G_2_		G_3_		G_4_	
	*F*	*M*	*F*	*M*	*F*	*M*	*F*	*M*
Birth weigth (g)	186.0 ± 3.4^a^	198.0 ± 4.4^a^	263.0 ± 3.02^b^	269.0 ± 3.3^b^	363.0 ± 3.01^c^	368.0 ± 3.9^c^	386.0 ± 3.8^d^	393.0 ± 5.2^d^
Expulsion interval (min)	67.2 ± 4.07^a,b^	81.1 ± 5.4^a^	65.3 ± 3.7^a,b^	68.5 ± 3.9^a,b^	61.3 ± 3.8^b^	59.4 ± 3.6^b^	55.4 ± 3.1^b^	54.7 ± 3.1^b^

Dams were divided into groups according to their weight: G_1_, 4–8 kg; G_2_, 8.1–16 kg; G_3_, 16.1–32 kg; G_4_, 32.1–39.6 kg. F, female; M, male. ^a,b.c,d^Different superscripts across columns indicate statistically significant differences between groups and according to sex (*p* < 0.05).

As shown in Table 5, two-way ANOVAs were used to assess all variables. *Post hoc* pairwise comparisons using Tukey HSD tests were used for analysis.

**Table 5:** Mean and standard error of the blood profile parameters of primiparous and multiparous dams classified according to weight.

Metabolites	G_1_		G_2_		G_3_		G_4_	
	*P*	*M*	*P*	*M*	*P*	*M*	*P*	*M*
Lactate (mg/dL)	7.02 ± 0.4^d^	5.46 ± 0.2^e^	7.80 ± 0.2^e^	5.24 ± 0.3^b,c,d^	8.74 ± 0.2^a,b^	7.22 ± 0.3^c,d^	9.83 ± 0.2^a^	8.44 ± 0.2^b,c^
Glucose (mm/dL)	74.8 ± 4.2^a,b^	88.0 ± 5.1^a^	73.6 ± 1.5^a,b^	85.6 ± 8.6^a^	72.3 ± 1.5^a,b^	82.3 ± 5.3^a,b^	63.9 ± 2.9^b^	70.3 ± 1.7^a,b^
Ca^2+^ (mmol/L)	1.97 ± 0.08^b,c^	1.76 ± 0.06^c,d^	2.05 ± 0.08^b,c^	1.64 ± 0.05^d^	2.12 ± 0.05^a,b^	1.92 ± 0.06^b,c,d^	2.42 ± 0.09^a^	2.11 ± 0.05^a,b^
pH	7.30 ± 0.01^a,b^	7.39 ± 0.01^a^	7.18 ± 0.04^c^	7.29 ± 0.01^a,b^	7.23 ± 0.015^b,c^	7.30 ± 0.01^a,b^	7.23 ± 0.02^b,c^	7.28 ± 0.01^b^
pO_2_ (mm/Hg)	21.1 ± 0.87^b,c^	26.1 ± 1.2^a^	18.6 ± 0.9^c^	24.0 ± 0.8^a,b^	20.9 ± 1.2^b,c^	24.3 ± 0.7^a,b^	14.0 ± 0.8^d^	17.3 ± 0.7^c,d^
pCO_2_ (mm/Hg)	52.7 ± 2.9^b^	47.0 ± 1.5^b^	57.9 ± 4.7^a,b^	47.8 ± 2.2^b^	56.8 ± 4.4^a,b^	48.4 ± 1.4^b^	69.4 ± 3.1^a^	60.0 ± 3.04^a,b^
HCO_3_^−^ (mmol/L)	19.5 ± 0.9^a,b,c^	22.7 ± 0.9^a^	17.8 ± 1.2^b,c^	20.8 ± 1.1^a,b^	16.3 ± 0.9^c^	18.7 ± 0.8^a,b,c^	15.6 ± 0.5^c^	18.2 ± 0.6^b,c^

Two-way ANOVA. Dams were divided into groups according to their weight: G_1_, 4–8 kg; G_2_, 8.1–16 kg; G_3_, 16.1–32 kg; G_4_, 32.1–39.6 kg. P, primiparous; M, multiparous. ^a,b.c,d^Different superscripts across columns indicate statistically significant differences between groups (*p* < 0.05).

As shown in Table 6, the effect of the sex of the puppy, birth weight, and expulsion interval on the likelihood of a puppy being stillborn was analyzed using a binary logistic regression model. The predictors were the sex of the puppy (male or female), birth weight (continuous), expulsion interval (continuous), and the interactions between sex and expulsion interval and between sex and birth weight. As the interaction between puppy sex and birth weight was not significant in the initial model, this was removed to increase fit. The probability distribution was “binomial” with a “logit” link function.

**Table 6:** Results of the final binary logistic regression model for factors affecting the likelihood of stillbirth.

Predictor	Estimate	Std. error	*z* value	Odd’s ratio	*p-*value
Intercept	−6.078996	1.064111	−5.713	0.0023	< 0.001
Birth weight	0.010371	0.002153	4.817	1.0104	**< 0.001**
Expulsion interval	−0.003644	0.011027	−0.330	0.9964	0.741
Sex male	−0.235426	0.780870	−0.301	0.7902	0.763
Expulsion interval: Sex Male	0.027298	0.012089	2.258	1.0277	**0.024**

Significant differences are indicated in bold letters.

As shown in Tables 7, 8, Pearson correlations were used to analyze correlations; as shown in Table 9, Spearman rank correlations were used because the variables did not have a normal distribution.

**Table 7:** Correlations between dam weight and uterine dynamics.

Variables	Correlation coefficient *(r)*	*p*-value
G_1_		
Contractions intensity (mmHg)	0.0261	0.904
Contractions duration (sec)	−0.265	0.210
Contractions number	−0.142	0.509
Interval between contractions (min)	0.164	0.443
G_2_		
Contractions intensity (mmHg)	−0.165	0.442
Contractions duration (sec)	−0.157	0.463
Contractions number	−0.234	0.270
Interval between contractions (min)	0.199	0.352
G_3_		
Contractions intensity (mmHg)	0.142	0.507
Contractions duration (sec)	0.122	0.570
Contractions number	0.147	0.493
Interval between contractions (min)	−0.162	0.450
G_4_		
Contractions intensity(mmHg)	−0.137	0.524
Contractions duration (sec)	−0.378	0.068
Contractions number	−0.140	0.514
Interval between contractions (min)	0.083	0.697

Pearson correlations. Dams were divided into groups according to their weight: G_1_, 4–8 kg; G_2_, 8.1–16 kg; G_3_, 16.1–32 kg; G_4_, 32.1–39.6 kg.

**Table 8:** Pearson correlations between uterine dynamics and blood profile.

Variables	Correlation coefficient *(r)*	*P*-value
Contractions intensity		
pH	0.007	0.949
PCO_2_ (mmHg)	0.006	0.954
PO_2_ (mmHg)	0.002	0.983
Glucose (mg/dL)	0.007	0.943
Ca^++^ (mmol/L)	0.047	0.646
Lactate (mmol/L)	−0.066	0.520
HCO_3_^−^	−0.007	0.946
Contraction duration		
**pH**	−0.250*	**0.014**
**PCO**_2_ **(mmHg)**	0.347*	**<0.001**
**PO**_2_ **(mmHg)**	−0.230*	**0.024**
**Glucose (mg/dL)**	−0.301*	**0.003**
**Ca** ^++^ **(mmol/L)**	0.365*	**<0.001**
**Lactate (mmol/L)**	0.380*	**<0.001**
**HCO**_3_^**−**^	−0.322*	**0.001**
**Contraction number**		
**pH**	−0.393*	**<0.001**
**PCO**_2_ **(mmHg)**	0.440**	**<0.001**
**PO**_2_ **(mmHg)**	−0.536**	**<0.001**
**Glucose (mg/dL)**	−0.362*	**<0.001**
**Ca** ^++^ **(mmol/L)**	0.619**	**<0.001**
**Lactate (mmol/L)**	0.741***	**<0.001**
**HCO**_3_^**−**^	−0.556**	**<0.001**

*Weak correlation; **moderate correlation; ***strong correlation. Values in bold represent significant correlations.

**Table 9:** Spearman correlations between uterine dynamics and dip 2.

Variables	Correlation coefficient *(r)*	*P*-value
Contractions intensity (mmHg)	0.119*	0.248
Contractions duration (sec)	0.179*	0.081
Contractions number	0.248*	**0.015**
Interval between contractions (min)	−0.248*	**0.015**

*Weak correlation. Values in bold represent significant correlations.

### Ethical statement

Before carrying out the study, informed consent was obtained from the animals’ owners, authorizing the procedures. All work was performed under Mexico’s Official Norm NOM-062-ZOO-1999 guidelines on the technical specifications for animal production, care, and ethical use in applied ethological studies (37). This project was approved by the Ph.D. Program in the Biological and Health Science Academic Committee (number CBS.114.19). All the female dogs evaluated in this study were treated gently, avoiding stress due to handling as much as possible; the use of an electronic fetal and uterine monitor greatly facilitated this aspect because it is not a painful or invasive technique.

## Results

As expected, larger bitches had a higher number of puppies per litter: G_1_ had an average of 3.3 puppies per litter, G_2_ had an average of 4.5 puppies per litter, G_3_ had an average of 5 puppies per litter, and G_4_ had an average of 8.9 puppies per litter. These numbers affected the other parameters.

It is important to mention that the FHR could not be evaluated in all fetuses because the fetal monitor could only perceive the heartbeats of some fetuses at random. Therefore, there was no way to know which frequency belonged to which fetus. However, all the puppies’ heart rates were evaluated after birth.

### Expulsion phase duration

There was no significant difference between primiparous and multiparous dams in the expulsion phase duration (*F*_1_ = 2.018, *p* = 0.159). However, there was a significant difference among weight groups (*F*_3_ = 9.963*, p* < 0.001). Tukey’s HSD tests showed that the expulsion phase duration was, on average, longer in dams from G_4_ (381.0 ± 22.0 min) compared to dams from the three other groups (G_1_: 247.0 ± 18.2 min, *p* < 0.001; G_2_: 307.0 ± 11.7 min, *p* = 0.02; G_3_: 302.0 ± 17.9, *p* = 0.009). There was a nonsignificant trend toward an interaction between weight group and parity (*F*_3_ = 2.515*, p* = 0.063) (Table 1).

### Expulsion interval between puppies

The average expulsion interval between puppies was significantly longer in primiparous dams (72.7 ± 2.06 min) than in multiparous dams (57.4 ± 1.67 min; F_1_ = 46.166, *p* < 0.001). The expulsion interval also differed significantly among weight groups (F_3_ = 13.673, *p* < 0.001). *Post hoc* comparisons showed that the expulsion interval was significantly longer in G_1_ (74.6 ± 3.27 min) than in G_3_ (61.5 ± 2.78 min*; p* < 0.001) and significantly shorter in G_4_ (55.5 ± 2.47 min) than in G_1_ (74.6 ± 3.27 min; *p* < 0.001) and G_2_ (68.8 ± 2.32 min; *p* < 0.001), as shown in Table 1.

### Stillborn puppies

The proportion of stillborn puppies was higher in primiparous dams (G_4_: 23.61%, G_3_: 13.88%, G_2_: 12.5, G_1_: 9.72%) than in multiparous dams (G_4_: 15.27%, G_3_: 9.72%, G_2_: 8.33%, G_1_: 6.94%; χ^2^ = 5.9811, df = 1, *p* = 0.014). There was no significant difference in the proportion of stillborn puppies among weight groups (χ^2^ = 0.66928, df = 3, *p* = 0.880) or primiparous and multiparous mothers classified according to weight groups (χ^2^ = 7.3018, df = 7, *p* = 0.398). The number of stillborn puppies from primiparous and multiparous dams classified according to their weight is shown in Table 1.

### Birth weight

The average birth weight was significantly higher in puppies from multiparous dams (308.0 ± 11.6 g) compared to primiparous dams (296.0 ± 12.2 g; *F*_1_ = 8.438, *p* = 0.005; Table 1).

### Intensity of contractions

The intensity of contractions was significantly higher in primiparous dams (30.4 ± 0.555 mmHg) than in multiparous dams (25.5 ± 0.507 mmHg; *F*_1_ = 96.360, *p* < 0.001). Contraction intensity differed significantly among weight groups (*F*_3_ = 41.073, *p* < 0.001). *Post hoc* comparisons showed that the intensity of contractions was significantly higher in G_1_ (32.6 ± 0.771 mmHg) than in G_2_ (27.5 ± 0.673 mmHg; *p* < 0.001), G_3_ (26.2 ± 0.620 mmHg*; p* < 0.001) and G_4_ (25.5 ± 0.766 mmHg; *p* < 0.001). Contraction intensity was also higher in G_2_ than in G_4_ (*p* = 0.025). The number, intensity, duration, and interval of contractions are shown in Table 2 for primiparous and multiparous bitches classified according to their weight.

### Duration of contractions

Contractions were significantly longer in primiparous dams (171.0 ± 3.97 s) than in multiparous dams (119.0 ± 2.24 s; *F*_1_ = 123.147, *p* < 0.001). There were no significant differences among weight groups (*F*_3_ = 0.255, *p* = 0.857) (Table 2).

### Number of contractions

The number of contractions was significantly higher in primiparous dams (16.6 ± 0.524) than in multiparous dams (13.0 ± 0.481; *F*_1_ = 156.10, *p* < 0.001). Likewise, the number of contractions differed significantly among weight groups (*F*_3_ = 160.78, *p* < 0.001). *Post hoc* tests revealed that G_4_ (18.7 ± 0.524) had significantly more contractions than the three other groups (G_1_: 10.5 ± 0.417, *p* < 0.001; G_2_: 13.0 ± 0. 483*, p* < 0.001; G_3_: 16.8 ± 0.454*, p* < 0.001). The number of contractions in G_3_ was significantly higher than that in G_1_ (*p* < 0.001) and G_2_ (*p* < 0.001). The number of contractions in G_2_ was significantly higher than that in G_1_ (*p* < 0.001) (Table 2).

### Interval between contractions

The general linear model (GLM) revealed that the interval between contractions was significantly longer in multiparous dams (5.71 ± 0.090 min) than in primiparous dams (4.39 ± 0.069; χ^2^ = 43.749, df = 1, *p* < 0.001). The interval between myometrial contractions also differed among weight groups (χ^2^ = 253.901, df = 3*, p* < 0.001); the interval was longer in lighter bitches and was significantly affected by the interaction between parity and weight group (χ^2^ = 14.760, df = 3, *p* = 0.002). *Post hoc* Tukey HSD test results are reported in Table 2.

### Late deceleration of fetal rate (dip 2)

The results of the GLM revealed that the proportion of fetuses showing dip 2 was significantly higher in primiparous dams (0.950 ± 0.146) than in multiparous dams (0.416 ± 0.089; χ^2^ = 10.433, df = 1, *p* = 0.001). No significant differences were observed among weight groups (χ^2^ = 2.658, df = 3, *p* = 0.447). dip 2 was not significantly affected by the interaction between weight group and parity (χ2 = 0.154, df = 3, *p* = 0.985). The number (and rate ± SE) of fetuses showing dip 2 in primiparous and multiparous bitches classified according to their weight is shown in Table 3.

### Bradycardia

All puppies were auscultated to determine whether they had bradycardia. The rate of puppies with AB bradycardia was significantly higher in litters from primiparous dams (1.069 ± 0.155) than in those from multiparous dams (0.527 ± 0.100; χ^2^ = 9.420, df = 1, *p* = 0.002). No significant differences were observed among weight groups (χ^2^ = 2.104, df = 3, *p* = 0.551). The interaction between weight group and parity did not significantly affect the rate of puppies with AB bradycardia (χ^2^ = 0.331, df = 3, *p* = 0.954). The number (and rate ± SE) of puppies with AB bradycardia in litters from primiparous and multiparous dams classified according to their weight is shown in Table 3.

### Cyanosis

The rate of puppies with cyanotic oral mucosa was significantly higher in litters from primiparous dams (1.307 ± 0.173) than in those from multiparous dams (0.656 ± 0.113; χ^2^ = 11.038, df = 1, *p* < 0.001). No significant differences were observed among weight groups (χ^2^ = 0.714, df = 3, *p* = 0.870). The rate of puppies with cyanotic oral mucosa was not significantly affected by the interaction between weight group and parity (χ^2^ = 0.113, df = 3, *p* = 0.990). The number (and rate ± SE) of puppies with cyanotic oral mucosa from primiparous and multiparous dams classified according to their weight is shown in Table 3.

### Hypothermia and adynamia

The number of hypothermic and adynamic puppies was significantly higher in litters from primiparous dams (1.486 ± 0.183) than in those from multiparous dams (0.847 ± 0.127; χ^2^ = 8.563, df = 1, *p* = 0.003). No significant differences were observed among weight groups (χ^2^ = 1.157, df = 3, *p* = 0.763). The proportion of hypothermic and adynamic puppies was not significantly affected by the interaction between weight group and parity (χ^2^ = 0.285, df = 3, *p* = 0.963). The number (and proportion ± SE) of bradycardic, cyanotic, hypothermic, and adynamic puppies from primiparous and multiparous bitches classified according to their weight is shown in Table 3.

### Comparison of birth weight between females and males

Newborn male puppies (329.0 ± 5.19 g) were significantly heavier than newborn females (314.0 ± 5.46 g; *F* = 5.232, df = 1, *p* = 0.023). Pairwise comparisons are reported in Table 4.

### Comparison of the expulsion interval between female and male puppies and dam weight

The expulsion interval significantly differed according to dam weight (*F* = 9.095, df = 3, *p* < 0.001). Expulsion intervals were significantly longer for puppies from G_1_ (74.2 ± 3.47 min) than for puppies from G_3_ (60.3 ± 2.63 min, *p* = 0.005) and G_4_ (55.0 ± 2.22, *p* < 0.001). Likewise, puppies from G_2_ (67.0 ± 2.70 min) had significantly longer expulsion intervals than those from G_4_ (55.0 ± 2.22 min, *p* = 0.005). No significant difference was found between male (63.1 ± 1.99 min) and female puppies (61.5 ± 1.84 min, *p* = 0.373).

Table 5 shows the differences in blood profiles between primiparous and multiparous bitches. The metabolites evaluated were lactate, glucose, Ca^++^, pH, pO_2_, pCO_2_, and HCO_3_^−^.

### Lactate

Primiparous dams had significantly higher lactate levels (8.35 ± 0.213 mg/dL) than multiparous dams (6.59 ± 0.237 mg/dL; *F*_1_ = 70.67, *p* < 0.001). Lactate levels significantly differed among weight groups (*F*_3_ = 41.59, *p* < 0.001). G_4_ (9.14 ± 0.229 mg/dL) had significantly higher lactate levels than the three other weight groups (G_1_: 6.24 ± 0.279 mg/dL, *p* < 0.001; G_2_: 6.52 ± 0.332 mg/dL, *p* < 0.001; G_3_: 7.98 ± 0.265 mg/dL, *p* < 0.001). Similarly, lactate was significantly higher in G_3_ than in G_1_ (*p* < 0.001) and G_2_ (*p* < 0.001).

### Glucose

Multiparous dams (81.6 ± 2.95 mg/dL) had significantly higher glucose levels than primiparous dams (71.2 ± 1.50 mg/dL; *F*_1_ = 10.536, *p* = 0.002). Glucose levels differed significantly among weight groups (*F*_3_ = 3.990, *p* = 0.01). G_4_ (67.1 ± 1.83 mg/dL) had significantly lower glucose levels than G_1_ (81.4 ± 3.54 mg/dL, *p* = 0.01) and G_2_ (79.6 ± 4.46 mg/dL, *p* = 0.03).

### Ca^++^

Ca levels were significantly higher in primiparous dams (2.14 ± 0.046 mmol/L) than in multiparous dams (1.86 ± 0.0385 mmol/L; *F*_1_ = 32.277, *p* < 0.001). ANOVA results revealed significant differences in Ca levels among weight groups (*F*_3_ = 15.170, *p* < 0.001). Ca levels were significantly higher in G_4_ (2.26 ± 0.061 mmol/L) than in G_1_ (1.86 ± 0.054 mmol/L, *p* < 0.001), G_2_ (1.85 ± 0.065 mmol/L, *p* < 0.001) and G_3_ (2.02 ± 0.047 mmol/L, *p* = 0.006).

### pH

Multiparous dams (7.32 ± 0.010) had significantly higher pH levels than primiparous dams (7.24 ± 0.015; *F*_1_ = 24.323, *p* < 0.001). Two-way ANOVA showed that pH levels differed significantly among weight groups (*F*_3_ = 24.323, *p* < 0.001). G_1_ (7.35 ± 0.015) had significantly higher pH levels than the three other groups (G_2_: 7.23 ± 0.0267, *p* < 0.001; G_3_: 7.27 ± 0.014, *p* = 0.005; G_4_: 7.25 ± 0.012, *p* < 0.001).

### pO_2_

Primiparous dams (18.7 ± 0.645 mm/Hg) had significantly lower pO_2_ levels than multiparous dams (22.9 ± 0.664 mm/Hg; *F*_1_ = 38.466, *p* < 0.001). pO_2_ levels also differed significantly among weight groups (*F*_3_ = 15.170, *p* < 0.001). Specifically, *post hoc* tests showed that G_4_ (15.6 ± 0.664 mm/Hg) had lower pO_2_ levels than G_1_ (23.6 ± 0.892 mm/Hg, *p* < 0.001), G_2_ (21.3 ± 0.857 mm/Hg, *p* < 0.001) and G_3_ (22.6 ± 0.819 mm/Hg, *p* < 0.001).

### pCO_2_

pCO_2_ levels were significantly higher in primiparous dams (59.2 ± 2.10 mm/Hg) than in multiparous dams (50.8 ± 1.31 mm/Hg; *F*_1_ = 14.054, *p* < 0.001). There was a significant difference in pCO_2_ levels among weight groups (*F*_3_ = 8.775, *p* < 0.001). G_4_ (64.7 ± 2.36 mm/Hg) had significantly higher pCO_2_ levels than the three other groups (G_1_: 49.8 ± 1.73 mm/Hg, *p* < 0.001; G_2_: 52.9 ± 2.77 mm/Hg, *p* = 0.002; G_3_: 52.6 ± 2.47 mm/Hg, *p* = 0.001).

### HCO_3_^−^

Multiparous dams (20.1 ± 0.506 mmol/L) had significantly higher levels of HCO_3_^−^ than primiparous dams (17.3 ± 0.514 mmol/L; F_1_ = 18.132, *p* < 0.001). HCO3- levels also differed significantly among weight groups (*F*_3_ = 18.132*, p* < 0.001). G_1_ (21.1 ± 0.744 mmol/L) had significantly higher HCO_3_^−^ levels than G_3_ (17.5 ± 0.658 mmol/L, *p* = 0.001) and G_4_ (16.9 ± 0.486 mmol/L, *p* < 0.001).

### Stillborn puppies

The likelihood of a stillbirth was significantly affected by birth weight (χ^2^ = 29.224, df = 1, *p* < 0.001). As birth weight increased, puppies were more likely to be stillborn. For every increment of 1 g in birth weight, puppies had 1.04% higher odds of being stillborn. Likewise, as the duration of the expulsion interval increased, male puppies were significantly more likely to be stillborn than female puppies (χ^2^ = 5.943, df = 1, *p* = 0.015). For every additional minute, male puppies had 2.77% higher odds of being stillborn than female puppies. The results of the final binary logistic regression model are reported in Table 6.

Pearson correlation analysis between uterine dynamic variables and dam weight was used to calculate the correlation coefficient in Tables 7, 8. Table 7 shows correlations between dam weight and uterine dynamics (there was no significant correlation when dividing the bitches into groups). Table 8 shows correlations between uterine dynamics and blood profiles. Significant correlations are shown in bold. Regarding the duration and number of contractions, there were negative correlations with pH, PO_2_, glucose, and HCO_3_, and there were positive correlations with PCO_2_, Ca^++^, and lactate. In both tables, weak correlations are marked with an asterisk, moderate correlations are marked with two asterisks, and strong correlations are marked with three asterisks following the classification of Schober et al. (38).

Table 9 shows the correlations between uterine dynamics and dip 2. Spearman rank correlations were used for analysis. Significant correlations are indicated in bold. There was a positive correlation of dip 2 with the number of contractions and a negative correlation of dip 2 with the interval between contractions (*p* = 0.015).

## Discussion

The results showed significant differences between primiparous and multiparous dams that not only affected their health but also affected the overall status of their newborn puppies. Although the dogs gave birth under similar conditions, this variable was not completely standardized because the births occurred in different clinics and hospitals. However, all the dogs were placed on foam mats to keep them comfortable and maintain similar temperatures on the floor where they gave birth.

Regarding fetal and uterine monitoring, in some cases, when the dams were nervous or moved too much, the tocodynamometer and transducer had to be repositioned, which created small pauses in the recording. However, most of the bitches allowed the monitor and bands to be placed without discomfort, and only a few (three bitches in G_1_) initially showed nervousness. However, after a few minutes, they calmed down and allowed monitoring. During the 60 min that the monitoring was carried out, an average of 5.3 myometrial contractions and two puppies were expelled, which is similar to previous records made by Davidson (8).

### Expulsion phase duration

Although no significant differences in the duration of the expulsion phase were found between primiparous and multiparous dams, this variable was influenced by the dam’s weight, with heavier bitches (from G_4_) exhibiting longer expulsion phases. A retrospective study on dystocia compared dogs according to their size and weight range: small (<12.7 kg), medium (12.7–20.5 kg), and large dogs (>20.5 kg) (18). Contrary to the results of the current study, no differences among dog weights were recorded. Similarly, Zonturlu and Kacar (39) did not find significant differences in the length of expulsion between German Shepherd (7.49 ± 2.44 h) and Labrador Retriever bitches (7.38 ± 1 h). However, the expulsion interval between puppies differed, with ranges of 20–415 min and 5–405 min, respectively, while Baqueiro-Espinosa et al. (40) found that the most extended whelping duration (369.73 min) was observed in dams of different breeds and parity (ranging from 0 to 4).

This study demonstrated that expulsion phase duration is positively associated with litter size since the average number of puppies born in each weight group is as follows: G_1_, 3.3 puppies per bitch; G_2_, 4.3 puppies; G_3_, five puppies; and G_4_, 6.9 puppies. Specifically, the larger the litter size was, the longer the expulsion phase duration. Another point that has been considered in some studies is the dysfunction of myometrial contractions in animals with higher weights, as observed in overweight animal models. In rats, females with high fat and high cholesterol levels exhibited asynchronous myometrial contractions and increased parturition duration (41). However, in this study, obese dams were not included. It is also important to consider that the number of contractions could influence these results, as the lighter-weight females in this study had a lower number of contractions, which could shorten the expulsion phase duration. However, these contractions were significantly more intense than those in G_4_. The fact that larger bitches (G_4_) tend to have larger litters and larger puppies (42, 43) than smaller bitches could be another essential factor to consider.

### Expulsion interval between puppies

Primiparous dams and those in G_1_ had the longest expulsion interval between puppies (82.2 ± 4.86 min). The average interval length is between 5 min and 2 h (44), while intervals of 12–16 h between the first and the last fetus are considered dystocia (45). Several studies have reported similar findings, and the increase in the interpup interval has been related to physiological exhaustion of the bitch, ineffective myometrial contractions (46), and the size of the litter (41, 42). In G_1_, the average expulsion interval between puppies was 74.16 min; in G_2_, it was 66.96 min; in G_3_, it was 60.30 min; and in G_4_, it was 55.01 min. Thus, the larger the size of the dog was, the shorter the expulsion interval between puppies, probably because these dogs had a higher number of contractions and a higher number of puppies (i.e., larger litter size). However, although the lighter primiparous bitches had the longest expulsion intervals, whelping was shorter due to the number of puppies, which is lower in small-sized dams than in large-sized ones. For example, in G_1_, the interval between puppies was 74.16 min. If the bitch was carrying an average of 3 (3.3) fetuses, whelping would take 148.3 min. In G_4_, the interval between puppies was 55.01 min. If the bitch was carrying an average of 7 (6.9) fetuses, whelping would take 330 min.

### Stillborn puppies

The risk of stillbirth is associated with parity, as shown in Münnich and Küchenmeister’s (47) study, which concluded that primiparous bitches more than 6 years old had the highest frequency of stillbirths (66.1%) and delayed whelping (3.8%). A similar result was obtained in the present study, where the proportion of stillbirths was higher in primiparous bitches in G_4._ Apart from the higher frequency of stillbirths, primiparous bitches have an increased risk of requiring C-sections (*p* = 0.004), and this is directly related to the presence of stillborn puppies (40). Some authors attribute this effect to the longer parturition duration and the lack of experience in primiparous bitches (48). Nonetheless, other reports indicate that parity is not related to stillbirths (49, 50), while other authors mention that dams only exhibit a constant rate of stillbirths after the fourth litter (51, 52).

Regarding the higher proportion of stillbirths in G_4_ bitches, maternal overweight is a risk factor for stillbirth in mammals (53). This is due to impaired placental function, which increases the stillborn risk (54). However, this factor was not related to the results of the present study, as overweight females were excluded.

### Birth weight

The positive relationship between the weight of the dam and the birth weight of the puppies observed in the current study has been recognized as a factor that might affect puppies’ development (55), and similar results have been reported in livestock. In lambs, the weight of the ewes significantly affected the birth weight of lambs due to maternal nutrition. In this sense, the quality and amount of nutrients obtained during gestation influence fetal growth (56). Another study assessing the influence of breed and average weight found that puppies of medium-sized breeds (10–20 kg) had 0.99 times lower perinatal mortality rates than large breeds (> 20 kg) (40). Regarding parity, the higher weight recorded in puppies born from multiparous dams in this study is different from the results of Tesi et al. (57) in toy and small-sized dogs, as parity did not affect puppies’ birth weight or neonatal mortality in that study. In contrast, lambs from primiparous ewes had the lowest weight (58).

### Intensity of contractions

In rats (41), bovines (59), and humans (60), obesity is related to the presentation of more intense uterine contractions, and this may be associated with the regulation of connexin-43 in myometrial myocytes. In the present study, we did not include obese bitches; however, the most intense uterine contractions occurred in primiparous bitches in G_1_ and G_2_ (smaller bitches) as well as multiparous bitches in G_3_ and G_4_ (larger bitches). Therefore, more intense uterine contractions could be associated not only with the weight of the dams, as in the primiparous and lighter bitches, but also with the weight of the newborns at birth and the size of the litter, with larger-sized bitches having larger litters (43, 61) and therefore having less space *in utero* as it is fully occupied by fetuses, as well as uterine fatigue in very prolonged parturitions (46).

### Duration of the contractions

In primiparous dams, the duration of contractions was more prolonged than that in multiparous dams, but their weight did not significantly affect the myometrial contraction time. The stress response is triggered in the first parturition, increasing circulating epinephrine levels, reducing the uterus’s contractile activity and increasing its duration (56). Likewise, when comparing multiparous and nulliparous women, primiparous patients had longer active labor and pushing phases (62); oxytocin and its action on uterine oxytocin receptors are required to promote strong and effective contractions (63).

### Number of contractions

Some breeds, such as Boxers, Border Collies, Labrador Retrievers, and Golden Retrievers, are predisposed to uterine inertia. Other predisposing factors include the dam’s age, disproportionately large or small litters, obesity, and hormonal or nutritional imbalances (42). In contrast to these findings regarding predisposed breeds, in this study, the heaviest (G_4_) primiparous bitches exhibited more uterine activity (more contractions) than multiparous bitches, and this group mainly consisted of Labradors and Golder Retrievers. This finding could explain why the expulsion interval between puppies was lower (55.01 min) on these bitches.

### Interval between contractions

The interval between contractions was greater for the lighter multiparous bitches, and primiparous bitches had more contractions; thus, the intervals between contractions in primiparous bitches are shorter than those in multiparous bitches. According to Olsson (64), the expulsion phase is triggered by the increase in plasma vasopressin concentration; in multiparous bitches, this hormone may decrease as the time of parturition increases.

### Blood profile

In general, the heaviest primiparous bitches presented the most critical changes in blood profiles. The greater the dam weight was, the larger the litter, the longer the labor, and the higher the incidence rates of uterine inertia and whelping complications. These results are associated with the longer whelping duration, which impairs uterine activity, and the consequent physiological ischaemia, hypoxia, and acidification (65) observed, with increased levels of lactate and pCO_2_ and a decrease in pO_2_ observed in G_4_. The elevations in glucose levels registered in heavier bitches are similar to those reported in bitches and puppies, where fetal dystocia induced an hyperglycaemic state, along with an increase in cortisol, a hormone known to mobilize glucose through glycogenolysis and gluconeogenesis (66). Therefore, the whelping complications reported in G_4_ bitches are consistent with the biochemical profile of dystocia cases.

### dip 2

There are few studies where dip 2 has been evaluated in bitches. Gilet al. (20) found that fetuses exhibited distress when the FHR was between 160 and 180 bpm for 60 s or more. Contrary to the findings of Gil et al. (20), who observed that both primiparous and multiparous dams could present fetuses with HR decelerations, we found that dip 2 developed in fetuses of 44 primiparous bitches and 23 multiparous bitches; thus, dip 2 presentation was more likely to occur in fetuses of primiparous bitches, and in 29 bitches of the total study population, dip 2 was not observed.

### Bradycardia

In some studies (67, 68), the welfare of canine fetuses has been evaluated based on fetal movements and heartbeat, and severe fetal distress was considered with an FHR <180 bpm. According to Gil et al. (20), the day of parturition can be predicted using the FHR, which could help provide a timely intervention and reduce animal losses. Studies carried out in humans by Hon and Hon et al. (69–71) revealed fetal heartbeat variations when administering exogenous oxytocin in the mother during delivery or when the mother exercised. In contrast to these previous studies, in the present study, no drug was administered to the bitches, nor were they subjected to any exercise or stress, so the results obtained could be closer to events in a normal whelping in bitches.

In this study, 79 newborn puppies presented bradycardia, and these decreases in heart rate were more evident in pups born to primiparous dams. This is likely because the most intense uterine contractions were observed in primiparous dams, which makes the presentation of dip 2 decelerations more likely.

### Cyanosis

The number of cyanotic puppies was higher in primiparous dams, possibly due to the complications and lack of experience reported in animals at the first parity. Fetal asphyxia, hypoxia, and cyanotic mucous membranes are indicators of low vitality scores in several domestic species (72, 73). Fetal asphyxia due to constant uterine contractions and umbilical cord blood vessel occlusion increases the whelping duration (74).

### Hypothermia and adynamia

Primiparous bitches, having little or no experience with parturition or maternal behavior, tend to be less skillful in the maternal care of their pups. These newborns are altricial and require the help of the dam to move and thermoregulate (1, 75) as they are unable to do so on their own; this aligns with what was found in this study, where 69 newborn puppies from primiparous dams exhibited adynamia and hypothermia compared to 47 newborn puppies from multiparous dams. In this sense, maternal experience influences their care of the offspring.

### Sex of the puppies

The relationship between higher birth weight and male sex is observed in different mammal species. This is attributed to sexual dimorphism, as males tend to be larger than females, as reported in newborn piglets (76). Moreover, as previously discussed, the body weight of the bitches also influences the birth weight of the newborns. Therefore, for dams with high body weights, it is important to consider the adverse effects on the mother and on the puppy’s growth and survival.

## Conclusion

Electronic fetal and uterine monitoring is a tool that should be implemented in bitches in all veterinary clinics, hospitals, and dog breeding sites to ensure the well-being of pregnant bitches and newborns, as well as to decrease the high rates of perinatal mortality in this species. It is a practical, noninvasive technique that is easy to use and accessible in most cases.

Weight can affect the vitality of newborns and the uterine dynamics of bitches, as weight groups differed in the frequency, intensity, and duration of myometrial contractions. The greater the weight of the bitches was, the more uterine dynamics changed, with the most intense and frequent contractions occurring in the heaviest primiparous dams. The expulsion interval between puppies was highest in the lightest primiparous dams and lowest in the heaviest multiparous dams. The duration of the expulsion phase, as well as the number of stillbirths, was greater in the heavier primiparous females. Similarly, the heaviest pups were born to the heaviest primiparous dams. The highest number of stillbirths (16) was observed in primiparous females of G_4_, with a total of 72 stillborn pups (23.61%).

Newborn male puppies were significantly heavier than newborn females, and birth weight also differed significantly according to dam weight group. However, no significant differences in the expulsion interval were found between female and male puppies. Thus, these findings suggest that the sex of the newborn does not influence its survival.

### Future directions

Veterinarians in the field of obstetrics of domestic canines and felines have several objectives: to increase the proportion of fetuses born alive, to minimize mothers’ morbidity and mortality, and to increase newborn survival during the first week of life. Electronic fetal and uterine monitoring is a tool in veterinary medicine that could facilitate perinatal care, thereby improving the survival of pups and the welfare of the animals, saving valuable time when making decisions of vital importance, and helping reduce production costs due to losses or deaths. The evaluation and correct interpretation of decelerations of the fetal heartbeat can indicate whether a bitch will require a cesarean section in a timely manner, and thereby reduce mortality rates. Although this tool has many advantages, some authors (77) caution that it is not helpful in preventing cerebral palsy and other neurodevelopmental disorders. Thus, several techniques that, when combined, provide the most complete fetal and maternal evaluation possible should be used (4). For example, MFE using cardiotocography, thermography (78, 79), evaluation of newborn vitality (APGAR) (6, 80–82), gasometry (83, 84), and evaluation of the morphology of the umbilical cord can be performed.

## Data availability statement

The original contributions presented in the study are included in the article/supplementary material, further inquiries can be directed to the corresponding author.

## Ethics statement

The animal studies were approved by Before carrying out the study, an informed consent was provided to the animals’ owners evaluated, so they authorized carrying out the procedures. All work was performed under Mexico’s Official Norm NOM-062-ZOO-1999 guidelines on the technical specifications for animal production, care, and ethical use in applied ethological studies (32). This project was approved by Ph.D. Program in the Biological and Health Science Academic Committee with number CBS.114.19. All the females evaluated in this study were treated gently, avoiding to the maximum the stress that manipulation could generate, and the fact that using an electronic fetal and uterine monitor greatly facilitated this aspect because it is a non-painful or invasive technique. The studies were conducted in accordance with the local legislation and institutional requirements. Written informed consent was obtained from the owners for the participation of their animals in this study.

## Author contributions

KL-G: Investigation, Methodology, Writing – original draft. JM-B: Project administration, Writing – review & editing. UB-E: Formal analysis, Methodology, Writing – review & editing. DV-G: Project administration, Resources, Writing – review & editing. AO-H: Methodology, Project administration, Writing – review & editing. IH-Á: Supervision, Writing – review & editing. PM-M: Methodology, Supervision, Writing – review & editing. AD-O: Investigation, Supervision, Writing – review & editing. DM-R: Conceptualization, Investigation, Project administration, Writing – original draft, Writing – review & editing.
